# Techniques of TIPS in the treatment of liver cirrhosis combined with incompletely occlusive main portal vein thrombosis

**DOI:** 10.1038/srep33069

**Published:** 2016-09-13

**Authors:** Mengfei Zhao, Zhendong Yue, Hongwei Zhao, Lei Wang, Zhenhua Fan, Fuliang He, Jiannan Yao, Xiaoqun Dong, Fuquan Liu

**Affiliations:** 1 Department of Interventional Therapy, Beijing Shijitan Hospital, Capital Medical University, Beijing 100038, China; 2 Chaoyang Hospital, Capital Medical University, Beijing 100020, China; 3 Department of Gastroenterology, Stephenson Cancer Center, Department of Internal Medicine, College of Medicine, The University of Oklahoma Health Sciences Center, USA

## Abstract

The patients of liver cirrhosis associated with portal vein thrombosis (PVT) can be effectively treated by transjugular intrahepatic portosystemic stent shunt (TIPS). Although the corresponding TIPS procedures have already performed on the patients to different types of PVT, the procedures are not specific and the relationship between different types of PVT and technical success rate of TIPS is unclear. What’s more, we aimed to explore the relationship between survival and vascular patency immediately after TIPS. 191 subjects underwent retrospective assessment. Appropriate TIPS procedures were performed based on our more specific classification. The overall success rate of TIPS was 95.8% (183/191). Success rate was significantly different between Grade II and Grade IV thrombosis (*χ*^2^ = 5.294, *P* = 0.021). The 1-, 2-, 3-, 4-and 5-year survival rates were 95.6%, 89.1%, 83.1%, 76.5% and 67.8%, respectively. The overall survival time of completely patent PV and incomplete patent PV immediately after TIPS was 57.05 ± 0.75 vs. 39.12 ± 2.64 months, respectively (*P* < 0.0001). We conclude that appropriate TIPS procedures and lower grade of PVT are essential for better technical success rate of TIPS. The patency of target vessels is important for survival.

PVT refers to thrombosis within the portal vein trunk, with or without thrombus extension to the intrahepatic portal vein branches, the splenic or mesenteric veins. The presence of PVT significantly changes the natural history of liver cirrhosis, because it not only greatly increases the incidence of variceal rebleeding and refractory ascites but also negatively influences the survival^1^. There are several ways of conventional treatment, such as liver transplantation, anticoagulation and intervention therapy, etc.^2^,^3^,^4^. However the insertion of TIPS might confer extra benefit. The basis of this is probably due to reducing the duration or risk of hypotension that is likely to be detrimental for patients with decompensated liver disease. Moreover, successful TIPS insertions can maintain the persistent portal vein patency, and avoid thrombus extension into the portal venous system^5^. Several clinical studies have reported that it is feasible for the insertion of TIPS in patients with PVT^6^,^7^. However, the appropriate TIPS procedures are not specific and the relationship between different types of PVT and technical success rate of TIPS is unclear. What’s more, we don’t know the relationship between the cumulative survival time and vascular patency immediately after TIPS. The aim of this study was to show the appropriate TIPS procedures for different types of PVT and investigate the questions above.

## Result

### Technique success

**Figure 1:**
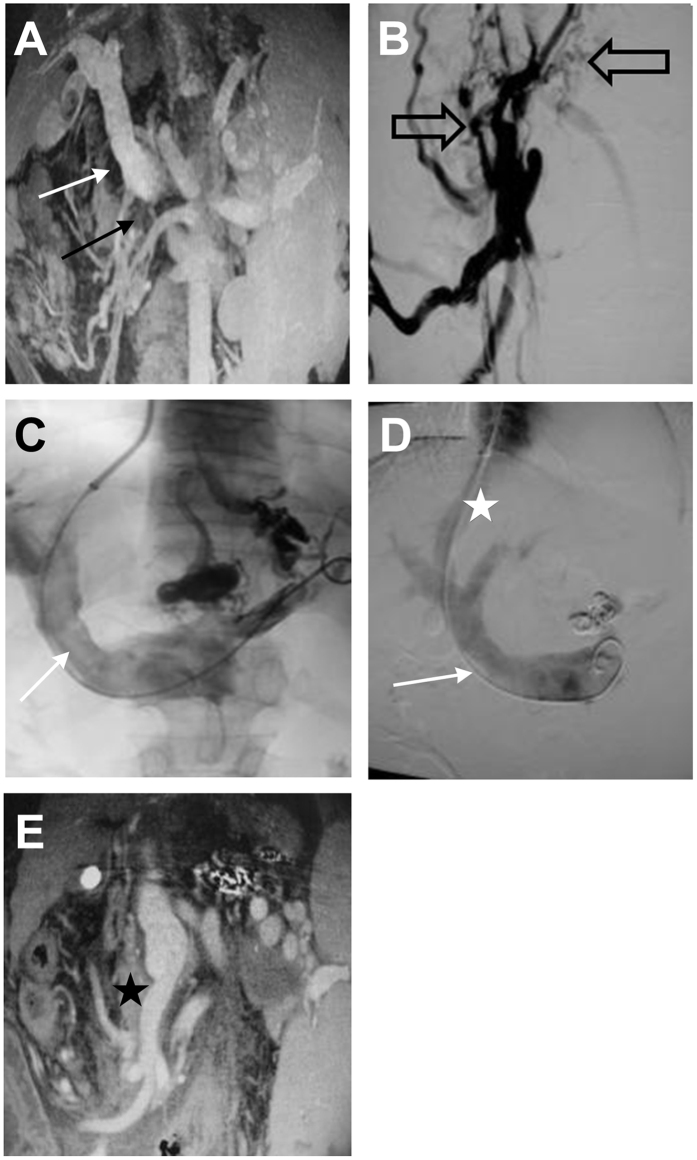
(**A–C**) Grade I main PVT (white arrows), Grade IV mesenteric vein (black arrows) and lateral branches (hollow arrows). (**D**) Grade I main PVT after TIPS (white arrows) with the shunt (white star). (**E**) One month after TIPS, the mesenteric vein thrombosis disappeared (black star).

**Figure 2:**
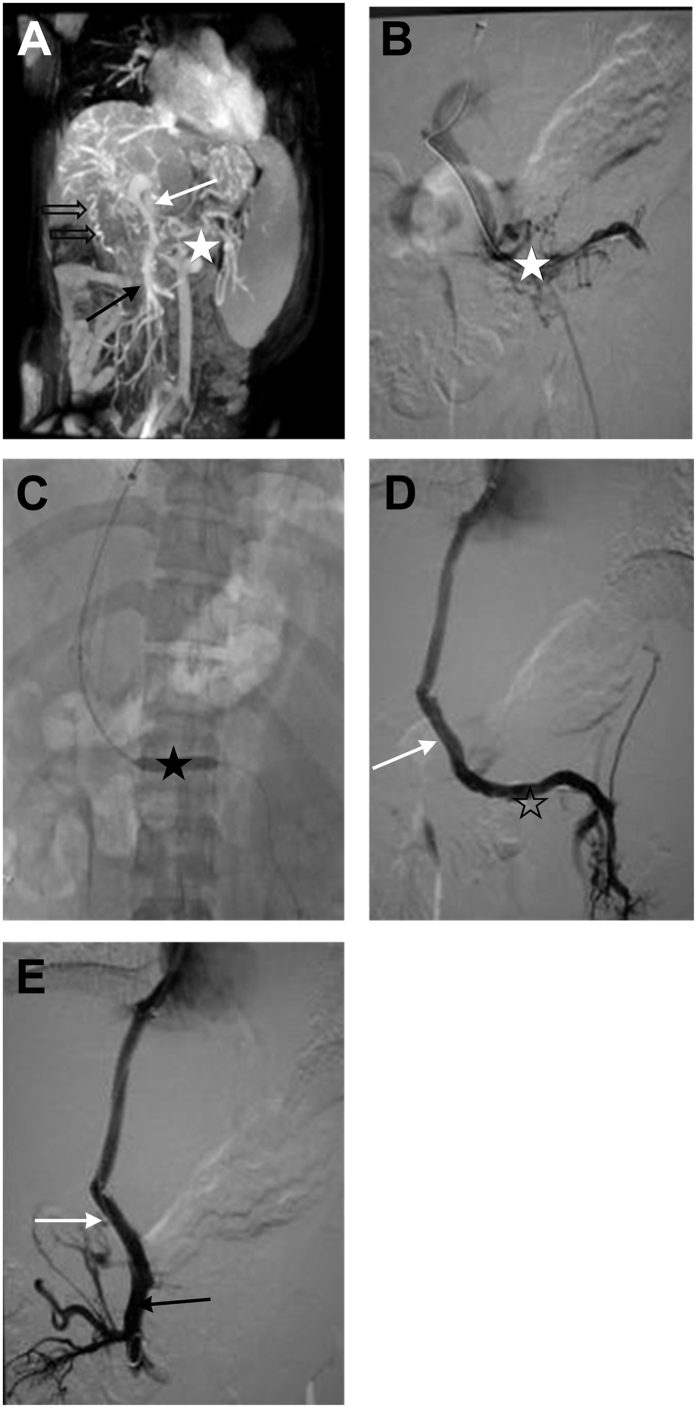
(**A,B**) Grade II main PVT (white arrows), lateral branches (hollow arrows), Grade 0 mesenteric thrombosis (black arrows), and Grade IV splenic vein thrombosis (white star). (**C**) Balloon dilation (black star). (**D,E**) After TIPS, the main PVT was reduced to Grade 0 (white arrows). The mesenteric vein was patent (black arrows), and the splenic vein thrombosis was Grade 0 (white star).

**Figure 3:**
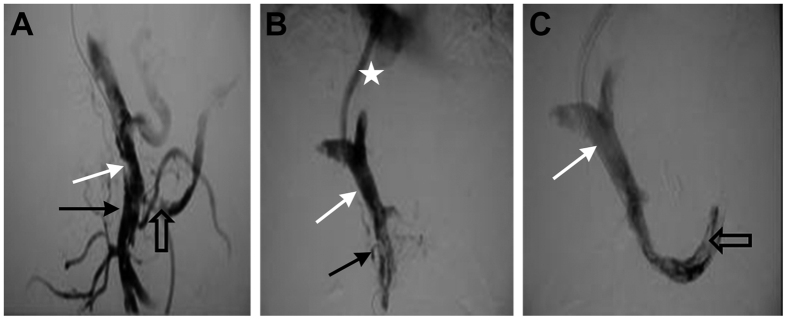
(**A**) Grade III main PVT (white arrows), Grade III mesenteric vein thrombosis (black arrows), and Grade IV splenic vein thrombosis (hollow arrows). (**B,C**) After TIPS, the main PVT was Grade I (white arrows), however, the mesenteric vein thrombosis remained as Grade III (black arrows). The splenic vein thrombosis was Grade III (hollow arrows) with the stunt (white arrows).

**Figure 4:**
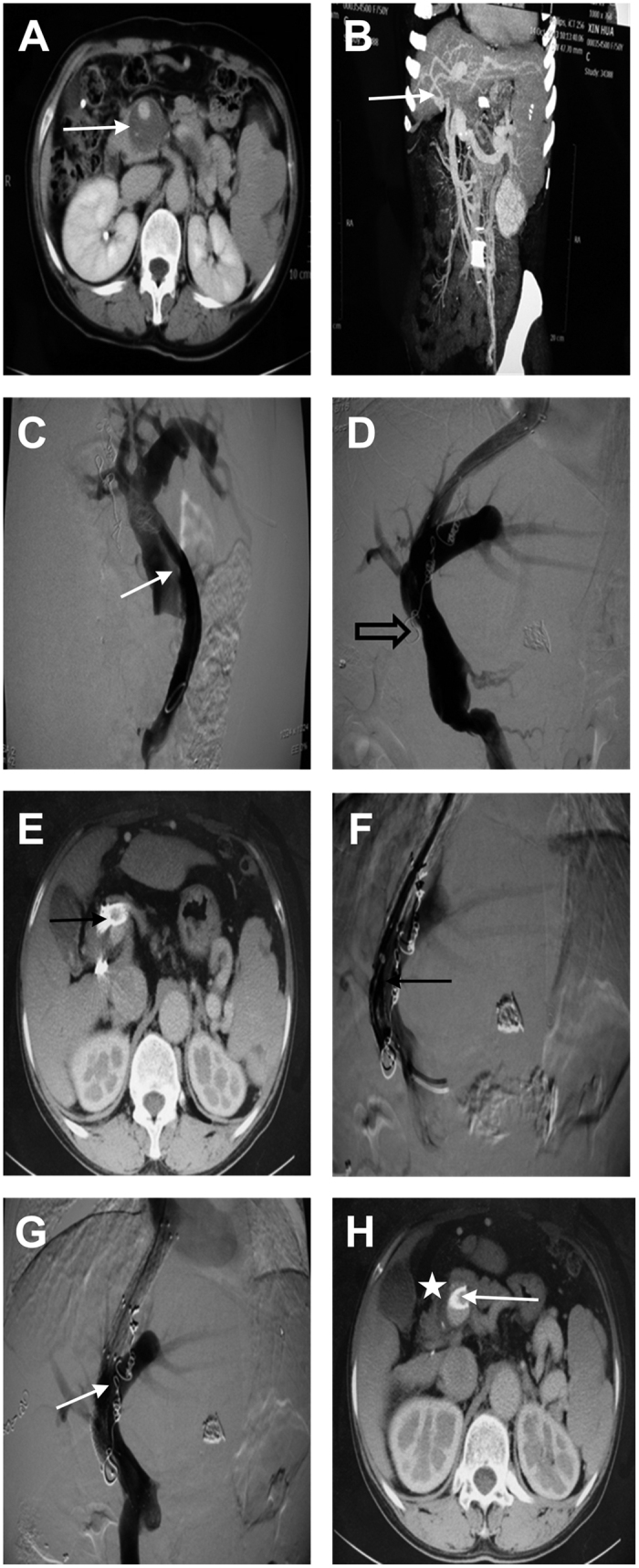
(**A–C**) Grade IV main PVT (white arrows). (**D**) Thrombosis (hollow arrows). (**E,F**) After TIPS, the distal stent had been embedded in the thrombosis (black arrows). (**G,H**) A bare stent was re-inserted (white arrows). There existed residual thrombosis (white star).

TIPS were successfully placed in 95.8% of patients (183/191). The success rate of Grade I (Fig. 1), Grade II (Fig. 2), Grade III (Fig. 3), and Grade IV (Fig. 4) of main PVT was 100.0% (38/38), 100.0% (54/54), 93.5% (43/46), and 93.8% (48/53), respectively. Success rate was significantly different between Grade II and Grade IV thrombosis (*χ*^2^ = 5.294, *P* = 0.021). The mean portal pressure was reduced from 32.9 ± 1.28 mmHg to 20.3 ± 0.9 mmHg (t = 105.44, *P* < 0.01). Among the 183 patients, 41 patients (22.4%) were treated with both percutaneous transhepatic portal technique (Fig. 3) and TIPS, while 27 patients (14.8%) were treated with indirect portal angiography and TIPS. Thrombolysis were removed either by indwelling the portal catheter or through local anti-coagulation in 9 patients (4.9%).

### Complications

Two (1.1%) patients suffered from severe complications during TIPS procedure: acute shunt occlusion in the first patient as the distal stent got embedded in the PVT (Fig. 4), whereas thoracic cavity bleeding in the second patient. During the 5 years since TIPS, 51 patients (27.9%) suffered from recurrent or emerging gastrointestinal bleeding, while 22 patients (12.0%) suffered from recurrent or emerging refractory ascites or hydrothorax. Hepatic encephalopathy was developed in 56 patients (30.6%). The thrombosis was completely disappeared in 93 patients (50.8%), and reduced in 81 patients (44.3%). However, the thrombosis either increased or recurred in 9 patients (4.9%). The overall shunt patency rate immediately after TIPS procedure was 91.3% (167/183) as shown in Figs 1, 2 and 4. The1-, 2-, 3-, 4-and 5-year primary shunt patency rate was 78.7%, 68.9%, 58.5% and 47.5%, respectively.

### Overall survival

The 1-, 2-, 3-, 4-and 5-year survival rate of patients was 95.6% (175/183), 89.1% (163/183), 83.1% (152/183), 76.5% (140/183), and 67.8% (124/183), respectively. The overall mean survival time was 51.46 ± 1.15 months (Supplementary Fig. 1).

### Vascular patency immediatly after TIPS and the survival of two groups

According to the angiography of PV immediately after TIPS procedure, the blood flow in the main portal vein was completely restored in 68.9% of the patients (126/183) (Fig. 2). The formation of thrombosis was significantly reduced in 27.9% of the patients (Fig. 4). The overall survival time of completely patent PV immediately after TIPS and incompletely patent PV immediately after TIPS was 57.05 ± 0.75 vs. 39.12 ± 2.64 months, respectively. Thus, according to Log-rank test, a significant difference in the survival time was observed (*P* < 0.0001) (Supplementary Fig. 2) (Table 1).

Among the 183 patients, 88 patients had PV combined with mesenteric vein thrombosis. Accroding to the angiography of developed mesenteric vein immediately after TIPS procedure, the blood flow was completely restored in 51.1% (45/88) of those patients (Fig. 1). However, no significant improvement of the blood flow was noted in 9.1% (8/88) of the patients after TIPS (Fig. 3). The survival time of completely patent mesenteric vein immediately after TIPS and incompletely patent mesenteric vein immediately after TIPS was 52.31 ± 1.37 vs. 42.33 ± 2.99 months, respectively (log-rank test, *P* = 0.004) (Supplementary Fig. 3).

Among the 183 patients, 36 patients had PV combined with splenic vein thrombosis. Accroding to the angiography of developed splenic vein immediately after TIPS procedure, the blood flow was completely restored in 58.3% (21/36) of those patients (Fig. 3). The formation of thrombosis was significantly reduced in 36.1% (13/36) of the patients. However, no significant improvement of the blood flow was noted in 5.6% (2/36) of the patients after TIPS. The survival time of completely patent splenic vein immediately after TIPS and incompletely patent splenic vein immediately after TIPS was 50.67 ± 2.63 vs. 32 ± 4.29 months, respectively (log-rank test, *P* = 0.007) (Supplementary Fig. 4).

Before and immediately after TIPS, the lumen occupancy (%) was 84 ± 7 and 20 ± 17 (t = 52.36, *P* < 0.05), respectively, in main portal vein; 65 ± 13 and 24 ± 16, respectively, in mesenteric vein (t = 10.288, *P* < 0.05); and 58 ± 7 and 29 ± 14, respectively, in splenic vein (t = 11.997, *P* < 0.05)(Table 1).

## Discussion

Liver cirrhosis is one of the important risk factors that promote the formation of PVT^8^. PVT may aggravate portal hypertension, which lead to variceal gastrointestinal bleeding and refractory ascites. These complications negatively influency the survival. In this study, all patients had developed PVT. All of the patients had portal hypertension, such as variceal gastrointestinal bleeding or refractory ascites or a combination of both above.

PVT was once considered to be a contraindication of TIPS. But as the improvement of the technology, more than 500 PVT patients have been reported to underwent TIPS until 2012^9^. Han G *et al*.^6^ had reported 75 subjects, whose success rate of TIPS was 75% (43/75). Angelo Luca *et al*.^7^ had reported 70 subjects, whose success rate of TIPS was 100%. As some studies reported, the degree of the PVT is an essential factor for the technical success^6^,^7^, and portal vein thrombosis with cavernous transformation usually cause the failure of TIPS^10^. Some scholars have classified the thrombosis in the portal vein system^7^,^10^,^11^. For example, Angelo Luca^7^ has classified PVT according to the proportion of thrombosis in the portal vein: Grade 0 invisible PVT; Grade I 1–25%, Grade II 26–50%, Grade III 51–75%, and Grade IV 76–100%. Most previous studies believe that conventional TIPS procedure can be directly applied to patients with images showing clear portal veins, while percutaneous transhepatic puncture combined with TIPS was applied in patients with images showing unclear portal veins. In this study, based on Angelo Luca’s grade of thrombosis, the thrombosis location was classified comprehensively mainly on the main portal vein thrombosis (Table 2). Based on this more specific classification, appropriate TIPS procedures were performed, leading to a high technical success rate of 95.8% (183/191). The technical success rate of TIPS for PVT in previous reports varied between 71–100%^6^,^7^. According to the results above, the technical success rate in our study is better. We also reported individual technical success rates of different types of PVT, and found there was statistically significant difference between Grade II and IV of the main PVT in TIPS success rates. We think the patients with lower grade of PVT have better technical success rate of TIPS.

Angelo *et al*.^7^ have reported that TIPS was conducted in 70 PVT patients, the preoperative lumen occupancy (%) was p 49 ± 28, m 37 ± 29, s 10 ± 21, respectively, while the postoperative lumen occupancy (%) was p 10 ± 18, m 10 ± 17, s 3 ± 11, respectively. Only 57.1% (40/70) of the patients achieved complete restoration of portal vein blood flow after TIPS, while PVT was significantly reduced in 30% (21/70) of the patients. However, nearly 12.9% (9/70) of PVT patients did not show any significant improvement after TIPS. As we all know, survival time is the most important factor for prognosis. In view of the research of Angelo above, we have a hypothesis, and that is whether vascular patency is an essential factor for cumulative survival time. After a TIPS procedure, we immediately performed angiography of PV, splenic and mesenteric veins to identify the patency of vessels. Patency of vessels conferred a better survival. Successful TIPS insertions can maintain the persistent portal vein patency, and avoid thrombus extension into the portal venous system^5^, which is perhaps the reason. Regarding the analysis of survival in case of mesenteric and splenic vein thrombosis, because some patients have different proportion of main portal vein thrombosis could be a competing effect for the survival analysis. Further investigation should be conducted to make the conclusion more convincing. I will make a cox regression analysis in the future.

Previous studies reported that TIPS has a mortality rate of 0–3%^12^. In this study, no body died because of TIPS, but two patients developed severe complications. The first one suffered from acute shunt occlusion because the distal stent was embedded in the PVT, so the shunt patency could be attained only after implanting the stent. The second patient got thoracic cavity bleeding when the percutaneous transhepatic stunt was implanted. The lateral and posterior costophrenic angle is deepened in patients with liver cirrhosis, and the puncture must have crossed the right diaphragm in this patient. Therefore, the bleeding occurred in the thoracic cavity due to negative pressure. The bleeding stopped when we performed emergency radiofrequency ablation of the puncture stunt. Although a combination of TIPS and percutaneous transhepatic portal puncture tends to improve the success rate of TIPS, the risks of thoracic and abdominal bleeding (seven cases have been described in our next article) should not be ignored.

Han *et al*.^6^ conducted a study on 57 patients, which included 35 cases of partial thrombosis (61.4%) and 22 cases of completely occlusive thrombosis (38.6%). The cumulative 1-year and 2-year shunt obstruction were 21% and 32%, respectively, while the incidences of hepatic encephalopathy were 25% and 27%, respectively. Moreover, the incidences of cumulative 1-year and 5-year variceal bleeding were 10% and 28%, respectively. Although we selected patients with incompletely occlusive thrombosis in the main portal veins, 48 cases (26.2%) suffered from Grade IV main PVT. Moreover, 31 cases were complicated as they had developed Grade IV mesenteric vein and/or splenic vein thrombosis. Therefore, the results are not only associated with thrombosis characteristics (such as degree, range, involved target vessels etc.), but they are also influenced by various parameters, such as liver function, technical procedures, postoperative treatment, symptoms relapse, thrombosis relapse, and follow-up time. We will make a cox regression analysis in the future.

In summary, we conclude that appropriate TIPS procedures and lower grade of PVT are essential for better technical success rate of TIPS. The patency of target vessels is important for survival.

## Materials and Methods

### Clinical data

This study has been approved by Institutional Review Board (IRB) committee at Beijing Shijitan Hospital. All procedures were conducted according to the guidelines approved by the ethics committee at Beijing Shijitan Hospital. Informed consent was acquired from each participate before the operation. This retrospective study was conducted at Department of Interventional Therapy in Beijing Shijitan Hospital. We analyzed the medical records of all patients with liver cirrhosis associated with incomplete occlusive thrombosis in the main portal vein who underwent TIPS between January 1997 to January 2010. Liver cirrhosis was diagnosed either by ultrasound and/or computed tomography (CT) and by clinical criteria for instance ascites or esophageal varices. PVT was was diagnosed either by contrast-enhanced CT and/or Magnetic Resonance Portal Vein (MRPV). In our study, the types of PVT were classified according to the location and severity of thrombosis (Table 2). Inclusion criteria were: (1) a definite diagnosis of PVT, what’s more, it should be incomplete occlusive thrombosis in the portal vein trunk, (2) TIPS is indicated in the treatment of recurrent variceal bleeding in patients who had failed to endoscopic and medical therapy, refractory ascites and hydrothorax, (3) the absence of malignancy, (4) the absence of previous primary thrombosis of the hepatic vessels, and (5) the absence of peptic ulcer, spontaneous bacterial peritonitis (SBP), and splenectomy (surgical shunt, devascularization, and splenectomy for the treatment of cirrhotic portal hypertension were not excluded). We excluded subjects who were unable to provide informed consent. In total, 183 patients (121 male, 62 female) were identified and included in the final analysis. On angiography, a reduction of thrombosis of PV, mesenteric or splenic veins by 50% was considered significant.

### Clinical parameters

The mean age of the subjects was 45.7 ± 12.4 years. The Child-Pugh score is calculated based on the five clinical and laboratory variables (serum total bilirubin, serum albumin, international normalised ratio (INR), ascites, encephalopathy). Preoperative Child-Pugh grade: A grade 23.5% (43/183), B grade 42.6% (78/183), and C grade 33.9% (62/183). The Model for end-stage liver disease (MELD) score is calculated based on the three laboratory variables (validated logarithmic index of serum bilirubin, creatinine and INR). The preoperative mean MELD score was 12.8 ± 5.7. The indications of TIPS are as follows: 159 cases developed recurrent varicealbleeding in patients who had failed to endoscopic and medical therapy, 29 cases developed refractory ascites and 10 cases developed hydrothorax and ascites (15 cases developed both gastrointestinal bleeding and ascites).

### TIPS procedure

TIPS placement technique: Different procedures were conducted according to the anatomic location and severity of PVT.

For a PVT of lower than Grade III, routine TIPS procedure was applied^13^. The hepatic vein (HV) was reached using a TIPS set (RUPS-100, Cook, Cook Inc., Bloomington, IL, United States), and the portal vein (PV) was punctured under the guiding of digital subtraction angiography. After the access to the right or left PV branch was achieved, we dilated the shunt with a balloon. In our study, bare stents (EV3, protégé; Cordis, Smart) were used in 50 patients before 2007. Fluency covered stents (Bard, Peripheral Vascular, Bard, Inc.) were used in 133 patients between 2007 to 2010. Once stents are deployed, trans-TIPS portal venography and pressure measurements in the main portal vein and the right atrium are repeated. The post-TIPS portosystemic pressure gradient is calculated. Markedly enlarged gastroesophageal collateral vessels were embolized with coils (Cook Incorporated, 750 Daniels Way Bloomington, IN).

For a PVT of Grade IV and obstructed intrahepatic portal branches, percutaneous transhepatic portal puncture was performed. Then a portal catheter was implanted, which was considered as the positioning marker for subsequent TIPS surgery.

For a PVT of >Grade III and patent intrahepatic portal branches, prior to the TIPS procedure, indirect portography was first conducted to identify the planar relationship between the lateral portal vein and puncture site.

If the stent was unable to cover the entire thrombosis, all the varicose veins were occluded; subsequently balloon dilatation, thrombectomy, and local thrombolysis were performed. The 4F pigtail catheter was indwelled into the distal portal vein thrombosis for 1–2 days; TIPS procedure was performed to implant the stent. for patients who developed gastrointestinal bleeding within one week and/or showed signs of intraoperative bleeding, injection of heparin saline was administered continuously through the indwelling catheter (500IU/h) for 1–2 days. For other patients, 500000–750000 unit urokinase was injected every day through this catheter for thrombolysis. Intermittently, injections of heparin saline 500 IU/h through this catheter was administered. Finally, TIPS procedure was performed to implant the stent.

After every TIPS procedure, we all immediately performed PV, splenic and mesenteric veins angiography to identify the patency of vessels. Warfarin was prescribed to all patients for at least one year to achieve an international normalized ratio (INR) of up to two times the upper limit of normal to prevent shunt dysfunction. To prevent hepatic encephalopathy in all patients, intravenous drip infusion of branched chain amino acids and oral lactulose were administered.

### Follow-up

The patients were followed for 5 years since TIPS or their death. Variceal bleeding, ascites, shunt patency, hepatic encephalopathy, and survival were assessed at 1, 3, 6 and 12 month and then yearly. Blood tests, coagulation function tests (prothrombin time, INR) and abdominal color Doppler ultrasound (CDUS) or CT scans. (The extension and degree of PVT and shunt), if possible, were obtained at the follow-up time points and any time when symptoms recurred (hematemesis, melena or large volume ascites). If the velocity of blood flow in the shunt was decreased or thrombosis was increased immediately after TIPS, TIPS dysfunction was defined.

### Statistical analysis

Numerical variables are expressed as mean value ± standard deviation. Normal continuous variables were compared using the Student’s *t* test. Non-normal continuous variables were compared using the Mann-Whitney ranksum test. Nominal variables are expressed as frequencies and compared using the *X*^*2*^ test or Fisher’s exact test. Accumulated proportions were assessed using Kaplan-Meier curves and compared using the log-rank test. All of the statistical analyses were performed with SPSS 17.0 (SPSS, Chicago, IL), and a two-tailed *P* value < 0.05 was considered statistically significant.

## Additional Information

**How to cite this article**: Zhao, M. *et al*. Techniques of TIPS in the treatment of liver cirrhosis combined with incompletely occlusive main portal vein thrombosis. *Sci. Rep.* **6**, 33069; doi: 10.1038/srep33069 (2016).

## Supplementary Material

Supplementary Information

## Figures and Tables

**Table 1:** Variation of thrombosis grading and lumen occupancy before and after TIPS^7^.

Thrombosis grading	Before TIPS	After TIPS	p value
P (No. of case)			
0	0	126	
I	37	30	
II	54	14	
III	44	12	
IV	48	1	
M (No. of case)			
0	95	166	
I	23	7	
II	16	4	
III	24	3	
IV	25	3	
S (No. of case)			
0	147	169	
I	16	7	
II	8	5	
III	6	2	
IV	6	0	
Lumen occupancy (%)			
P (portal vein)	84 ± 7	20 ± 17	0.012
M (mesenteric vein)	65 ± 13	24 ± 16	0.034
S (splenic vein)	58 ± 7	29 ± 14	0.029

p, MPV (main portal vein); m, mesenteric vein; s, splenic vein.

**Table 2:** Anatomic location and severity of portal vein thrombosis.

Anatomic location/type	No. of cases	P				M				S			
		I	II	III	IV	I	II	III	IV	I	II	III	IV
P	38	11	13	5	9	0	0	0	0	0	0	0	0
pb	41	8	16	7	10	0	0	0	0	0	0	0	0
pbm	29	3	6	12	9	7	4	5	13	0	0	0	0
pbs	6	2	1	1	2	0	0	0	0	3	1	1	1
pbms	15	2	5	4	4	5	7	1	2	10	2	1	2
pm	39	9	8	13	9	11	3	16	9	0	0	0	0
ps	10	1	5	1	3	0	0	0	0	2	4	2	2
pms	5	1	0	1	3	0	2	2	1	1	1	2	1
Sum	183	37	54	44	48	23	16	24	25	16	8	6	6

Note: p, MPV (main portal vein); m, mesenteric vein; s, splenic vein; pb (main portal vein+branch); pbm (main portal vein+branch+mesenteric vein); pbs (main portal vein+branch+splenic vein); pbms (main portal vein+branch+mesenteric vein+splenic vein); pm (main portal vein+mesenteric vein); ps (main portal vein+splenic vein); pms (main portal vein+mesenteric vein+splenic vein).

Severity of thrombosis was estimated as follows: Grade 0 (no detectable thrombus), Grade I (1–25% luminal occlusion), Grade II (26–50% luminal occlusion), Grade III (51–75% luminal occlusion), and Grade IV (76–100% luminal occlusion).
