# Supplementary material for: Techniques of TIPS in the treatment of liver cirrhosis combined with incompletely occlusive main portal vein thrombosis
Source: Sci Rep. 2016 Sep 13;6:33069. doi: 10.1038/srep33069 (PMC5020493; doi:10.1038/srep33069)
Supplement: Supplementary Information [file srep33069-s1.pdf]

Techniques of TIPS in the treatment of liver cirrhosis combined with incompletely occlusive main portal vein thrombosis

Mengfei Zhao, Zhendong Yue, Hongwei Zhao, Lei Wang, Zhenhua Fan, Fuliang He, Jiannan Yao, Xiaoqun Dong, Fuquan Liu

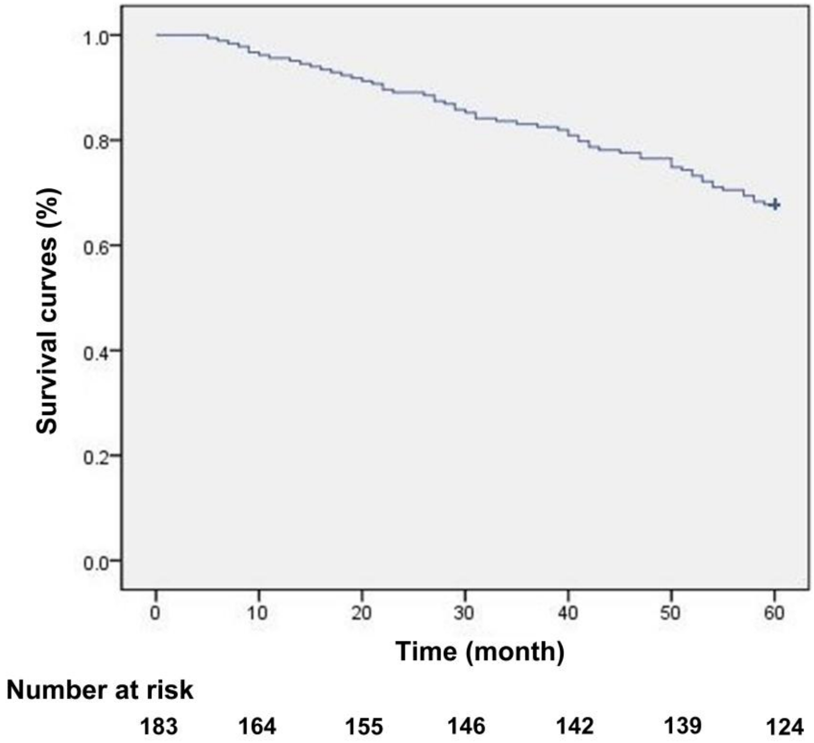

Supplementary Figure 1. Effects of TIPS on overall survival (5-year follow-up) of patients with PVT

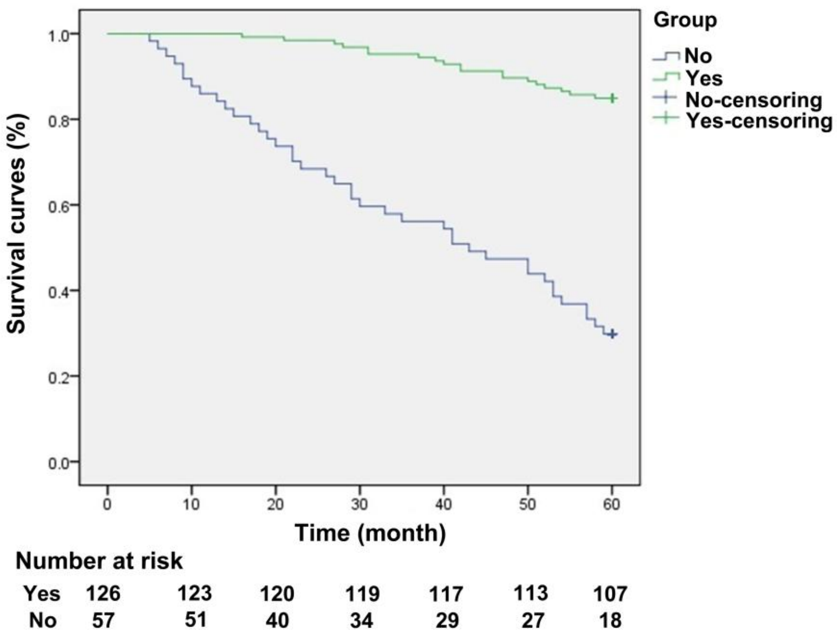

Supplementary Figure 2. Effects of completely patent PV immediately after TIPS (Yes) vs. incompletely patent PV immediately after TIPS (No) on overall survival (5-year follow-up) of patients with PVT

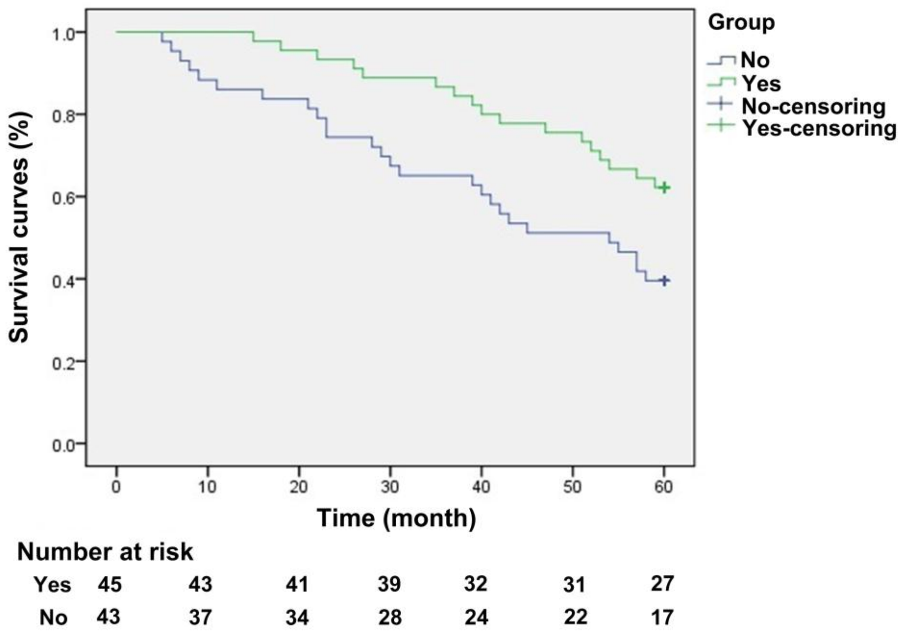

Supplementary Figure 3. Effects of completely patent mesenteric veins immediately after TIPS (Yes) vs. incompletely patent mesenteric veins immediately after TIPS (No) on overall survival (5-year follow-up) of patients with PVT

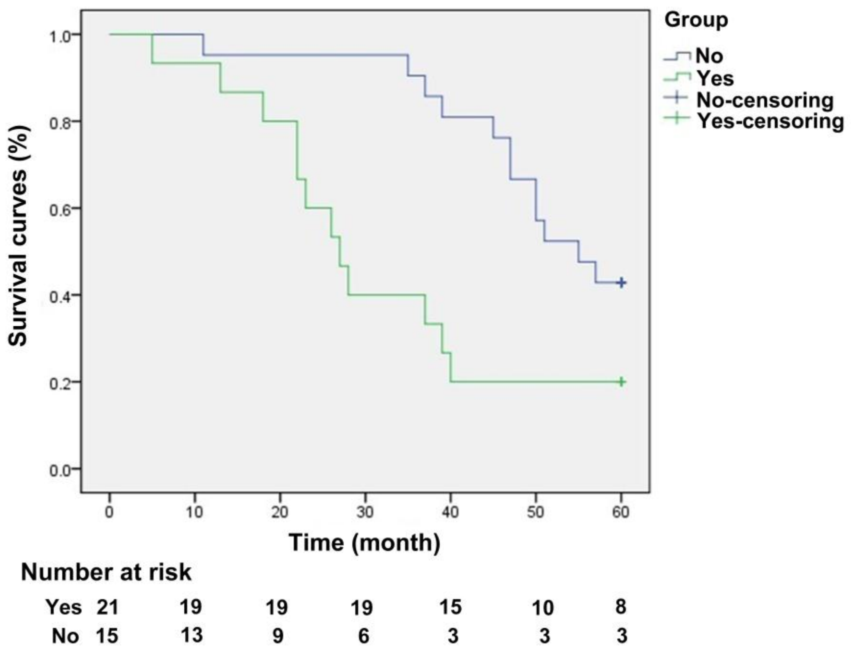

Supplementary Figure 4. Effects of completely patent splenic veins immediately after TIPS (Yes) vs. incompletely patent splenic veins immediately after TIPS (No) on overall survival (5-year follow-up) of patients with PVT
